# Irritable Bowel Syndrome Is Associated with an Increased Risk of Dementia: A Nationwide Population-Based Study

**DOI:** 10.1371/journal.pone.0144589

**Published:** 2016-01-05

**Authors:** Chien-Hua Chen, Cheng-Li Lin, Chia-Hung Kao

**Affiliations:** 1 Digestive Disease Center, Show-Chwan Memorial Hospital, Changhua, Taiwan; 2 Hungkuang University, Taichung, Taiwan; 3 Meiho University of Technology, Pingtung, Taiwan; 4 Management Office for Health Data, China Medical University Hospital, Taichung, Taiwan; 5 College of Medicine, China Medical University, Taichung, Taiwan; 6 Graduate Institute of Clinical Medical Science, School of Medicine, College of Medicine, China Medical University, Taichung, Taiwan; 7 Department of Nuclear Medicine and PET Center, China Medical University Hospital, Taichung, Taiwan; Charité-Universitätsmedizin Berlin, Campus Benjamin Franklin, GERMANY

## Abstract

**Purpose:**

Abnormal interaction in the brain–gut axis has emerged as one of the relevant pathophysiological mechanisms for the development of irritable bowel syndrome (IBS). Moreover, the brain–gut axis has recently been demonstrated to be crucial for the maintenance of cognitive performance. Therefore, we assessed the risk of dementia following diagnosis of IBS.

**Methods:**

Using the Taiwan National Health Insurance Research Database (NHIRD) to obtain medical claims data from 2000 to 2011, we employed a random sampling method to enroll32 298 adult patients with IBS and frequency-matched them according to sex, age, and baseline year with 129 192 patients without IBS.

**Results:**

The patients with IBS exhibited an increased risk of dementia [adjusted hazard ratio (aHR) = 1.26, 95% confidence interval (CI) = 1.17–1.35]after adjustment for age, sex, diabetes, hypertension, stroke, coronary artery disease (CAD), head injury, depression, and epilepsy, and the overall incidence of dementia for the cohorts with and without IBS was 4.86 and 3.41 per 1000 person-years, respectively. IBS was associated with an increased risk of dementia in patients older than 50 years in both male and female, and in those with comorbidity or without comorbidity. After adjustment for age, sex, and comorbidity, patients with IBS were also more likely to develop either non- Alzheimer’s disease (AD) dementia (aHR = 1.24, 95% CI = 1.15–1.33) or AD (aHR = 1.76, 95% CI = 1.28–2.43).

**Conclusions:**

IBS is associated with an increased risk of dementia, and this effect is obvious only in patients who are ≥50 years old.

## Introduction

Irritable bowel syndrome (IBS) is characterized by recurrent abdominal pain or discomfort with altered visceral hypersensitivity or gastrointestinal (GI) motility. Evidence supports that a diagnosis of IBS can be confidently made for the patients fitting the symptoms-based criteria and having no concerning features for organic diseases, including symptom onset after age 50, severe or progressively worsening symptoms, unexplained weight loss, nocturnal diarrhea, GI bleeding, unexplained iron-deficiency anemia, and family history of colonic cancer, celiac disease, or inflammatory bowel disease [1]. Some organic changes with altered inflammatory markers, dysbiosis, and genotypic expressions of inflammatory or neurotransmitter receptor molecules indeed can be observed in the IBS patients by employing more sensitive methods [2]. However, most IBS patients will have a negative evaluation result if they have no concerning features. IBS is a common functional GI disorder worldwide, and it accounts for approximately 11%–22.1% of cases in GI outpatient departments for Chinese populations [3,4]. Patients with IBS have greater risk for comorbidities, either constitutional or mental illness, resulting in a decline in quality of life and increase in medical expenditure [5–7]. The microbiome—brain—gut axis is composed of the central nervous system, neuroendocrine and neuroimmune systems, autonomic nervous system, enteric nervous system (ENS), and gut microbiome [8]. The brain can influence motility, sensation, and secretion in the GI tract via signaling between neurons, hormones, and cytokines. Moreover, the GI tract can modulate brain function reciprocally through the same mechanism [8, 9].

Dementia can be classified into non-Alzheimer’s disease (AD) dementia and AD. The etiological factors of dementia remain undetermined despite genetic, vascular, and psychological disorders being implicated as possible risk factors. AD is the most common pattern of dementia, and most patients with AD are older than 65 years. AD exhibits the characteristics of insidious onset and a clear-cut history of worsening cognition without substantial concomitant cerebrovascular disease. In addition to the criteria for dementia, the certainty of AD can be enhanced in the presence of pathophysiological processes such as biomarkers of brain amyloid-beta protein deposition, downstream neuronal degeneration, or injury. Dementia can cause cognitive or behavioral symptoms such as functional interference at work or during usual activities, and decline from previous levels of functioning and performing without delirium or major psychiatric disorders [10]. Dementia has a substantial impact on patients and their family, exerting a considerable burden on both direct and indirect social expenditures.

To assess the association between IBS and the subsequent development of dementia, this study adopted a nationwide population-based cohort design to analyze data from the National Health Insurance Research Database (NHIRD) in Taiwan.

## Methods

### Data Source

This study was conducted using data from the Longitudinal Health Insurance Database 2000 (LHID2000), which contains the original claims data of 1 000 000 randomly sampled persons from the 2000 Registry for Beneficiaries (23.75 million citizens). The National Health Insurance (NHI) program is a nationwide single-payer insurance system that was established in March 1995 and has covered nearly 99% of the population of people in Taiwan [11]. The electronic medical files contain details on utilities and health care services provided to individual patients, including demographic characteristics, complete outpatient visits, hospital admissions, International Classification of Diseases, 9th Revision, Clinical Modification (ICD-9-CM) diagnostic codes, prescriptions, and clinical orders(such as surgery). To comply with data privacy regulations, personally identifiable information is encrypted and all data are depersonalized.

### Sampled Participants

Using the LHID2000, we identified patients aged ≥20 years with newly diagnosed IBS (ICD-9-CM Code 564.1) between 2000 and 2011. The index date was set as the date of initial diagnosis of IBS. A non-IBS comparison cohort was randomly selected among patients without a diagnosis of IBS during 2000–2011, and these patients were frequency-matched with the patients with IBS at a ratio of 1:4according to age group (every 5 years), sex, and index year. The index date for the comparison cohort was randomly appointed a month and day with the same index year of the matched IBS cases. Patients with a history of dementia (ICD-9-CMCodes 290, 294.1, and 331.0) or with missing medical information were excluded from both cohorts.

### Outcome and Comorbidities

We identified the first diagnosis of dementia (ICD-9-CM Codes 290, 294.1, and 331.0) as the study end point. All cases were followed from the index date until the occurrence of dementia or December 31, 2011, and observations on the last date were regarded as censored observations. We defined baseline comorbidities, namely diabetes (ICD-9-CMCode250), hypertension (ICD-9-CMCodes401-405), stroke (ICD-9-CMCodes430-438), coronary artery disease(CAD) (ICD-9-CMCodes410-414), head injury (ICD-9-CMCodes850-854 and 959.01), depression (ICD-9-CMCodes296.2, 296.3, 300.4, and 311), and epilepsy (ICD-9-CMCode345).

### Ethics Statement

The National Health Research Institute (NHRI) encrypts patient personal information to protect privacy and provides researchers with anonymous identification numbers associated with relevant claims information, including sex, date of birth, medical services received, and prescriptions. Patient consent is not required to access the NHIRD. This study was approved to be exempted by the Institutional Review Board (IRB) of China Medical University and Hospital (CMUH104-REC2-115). The IRB specifically waived the consent requirement.

### Data Availability Statement

All data and related metadata were deposited in an appropriate public repository. The data on the study population that were obtained from the NHIRD (http://w3.nhri.org.tw/nhird//date_01.html) are maintained in the NHIRD (http://nhird.nhri.org.tw/). The NHRI is a nonprofit foundation established by the government. The use of the data needs the assessment and agreement by NHRI.

### Statistical Analysis

The distribution of age (20–34, 35–49, 50–64, and ≥65 years), sex, and comorbidity were compared between the two cohorts by using the chi-squared test for the categorical variables and the Student’s *t*test for the continuous variables. Cumulative incidence curves for dementia were plotted using the Kaplan—Meier method, and the differences in cumulative incidence between the two cohorts were tested using a log rank test. Dementia incidence densities were estimated by dividing the number of dementiacases by the number of person-years in each risk factor, and then stratified by age, sex, and comorbidity. Univariateand multivariate Cox proportion hazard regression models were employed to examine the effect of IBS on the risk of dementia, expressed as hazard ratios (HRs) with 95% confidence intervals (CIs). The multivariate Cox models were adjusted for age, sex, and comorbidities of diabetes, hypertension, stroke, CAD, head injury, depression, and epilepsy. When the patients were stratified according to age, sex, and comorbidity, the relative risk of dementia in the IBS cohort compared with the non-IBS cohort was also analyzed using Cox models. All analyses were performed using SAS Version 9.4 (SAS Institute, Cary, NC, USA). The significance level was set at less than 0.05 for the two-tailed P value.

## Results

**Fig 1:**
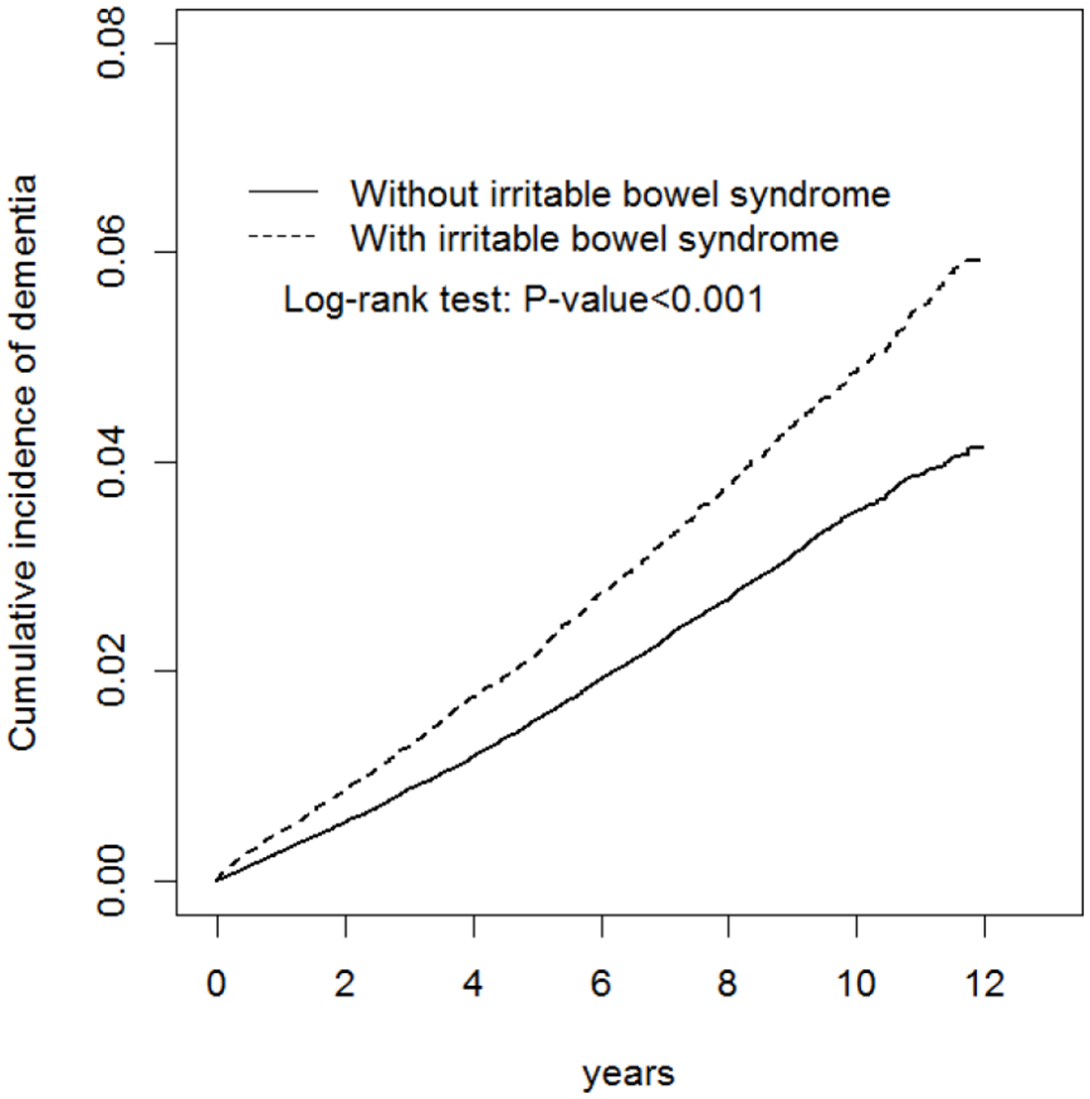
Cummulative incidence comparison of dementia for patients with (dashed line) or without (solid line) Irritable bowel syndrome.

Table 1 shows the demographic characteristics and comorbidities in cohorts with and without IBS. The study examined a cohort of 32 298 patients with IBS and a non-IBS cohort of 129 192 patients. Both cohorts had similar age and sex distributions, and were predominantly ≤49 years old (48.7%) and female (52.6%). The mean agesof the IBS and non-IBS cohorts were 51.5 [standard deviation (SD) = 16.4]and 51.0 (SD = 16.6) years, respectively. Compared with the non-IBS cohort, the IBS cohort exhibited higher prevalence in all comorbidities at the baseline (p < 0.001). During the mean follow-up of 6.89 years for the IBS cohort and 6.78 years for the non-IBS cohort, the results of Kaplan—Meier analysis showed that the IBS cohort exhibited a higher cumulative incidence of dementia (log-rank test, p < 0.001) (Fig 1).

**Table 1:** Demographic characteristics and comorbidities in cohorts with and without irritable bowel syndrome.

Variable	Irritable bowel syndrome		*p*-value
	No	Yes	
	N = 129192	N = 32298	
**Age, year**			0.99
≤ 34	23220(18.0)	5805(18.0)	
35–49	39600(30.7)	9900(30.7)	
50–64	35384(27.4)	8846(27.4)	
65+	30988(24.0)	7747(24.0)	
**Mean±SD**^†^	51.0(16.6)	51.5(16.4)	0.001
**Sex**			0.99
Female	67976(52.6)	16994(52.6)	
Male	61216(47.4)	15304(47.4)	
**Comorbidity**			
Diabetes	9872(7.64)	3084(9.55)	<0.001
Hypertension	35524(27.5)	11329(35.1)	<0.001
Stroke	4014(3.11)	1142(3.54)	<0.001
CAD	15941(12.3)	6877(21.3)	<0.001
Head injury	2549(1.97)	926(2.87)	<0.001
Depression	3621(2.80)	2852(8.83)	<0.001
Epilepsy	805(0.62)	272(0.84)	<0.001

Chi-Square Test;

^†^: T-Test

CAD denotes coronary artery disease

Table 2 shows the incidence and HR for dementia and dementia-associated risk factors. The overall incidence of dementia for the cohorts with and without IBS was 4.86 and 3.41 per 1000 person-years, respectively. After adjustment for age, sex, and comorbidities of diabetes, hypertension, stroke, CAD, head injury, depression, and epilepsy, the patients with IBS exhibited an increased risk of dementia relative to those without IBS[adjusted HR(aHR) = 1.26, 95% CI = 1.17–1.35]. The dementia incidence increased with age and tended to coincide with comorbidity incidence. Compared with patients who were ≤49 years old, the risk of dementia was 13.0-fold higher in those aged 50–64 years (95% CI = 10.2–16.7), and 85.5-fold higher in those aged ≥65 years (95% CI = 67.4–108.5). Besides, the multivariate models also showed that dementia was independently associated with each comorbidity, such as diabetes (aHR = 1.27, 95% CI = 1.17–1.37), hypertension (aHR = 1.41, 95% CI = 1.30–1.51), stroke (aHR = 1.65, 95% CI = 1.49–1.83), CAD (aHR = 1.22, 95% CI = 1.14–1.30), head injury (aHR = 1.61, 95% CI = 1.37–1.88), depression (aHR = 1.77, 95% CI = 1.58–1.98), and epilepsy (aHR = 1.88, 95% CI = 1.48–2.38).

**Table 2:** Incidence and Hazard ratio for dementia and dementia-associated risk factors.

Variable	Event	PY	Rate	Crude HR(95% CI)	Adjusted HR(95% CI)
**Irritable bowel syndrome**					
No	2981	875387	3.41	1.00	1.00
Yes	1081	222562	4.86	1.43(1.33, 1.53)***	1.26(1.17, 1.35)***
**Age, year**					
≤ 49	72	568819	0.13	1.00	1.00
50–64	599	302819	1.98	15.8(12.4, 20.2)***	13.0(10.2, 16.7)***
65+	3391	226312	15.0	122.9(97.3, 155.3)***	85.5(67.4, 108.5)***
**Sex**					
Female	2106	591128	3.56	1.00	1.00
Male	1956	506822	3.86	1.09(1.02, 1.16)**	0.98(0.92,1.05)
**Comorbidity**					
**Diabetes**					
No	3289	1025444	3.21	1.00	1.00
Yes	773	72506	10.7	3.42(3.16, 3.70)***	1.27(1.17,1.37)***
**Hypertension**					
No	1259	806169	1.56	1.00	1.00
Yes	2803	291780	9.61	6.26(5.85, 6.69)***	1.41(1.30, 1.51)***
**Stroke**					
No	3592	1073601	3.35	1.00	1.00
Yes	470	24349	19.3	6.03(5.47, 6.64)***	1.65(1.49, 1.83)***
**CAD**					
No	2440	958073	2.55	1.00	1.00
Yes	1622	139877	11.6	4.63(4.35, 4.93)***	1.22(1.14,1.30)***
**Head injury**					
No	3897	1078854	3.61	1.00	1.00
Yes	165	19095	8.64	2.46(2.10, 2.87)***	1.61(1.37, 1.88)***
**Depression**					
No	3715	1060029	3.50	1.00	1.00
Yes	347	37921	9.15	2.67(2.39, 2.98)***	1.77(1.58, 1.98)***
**Epilepsy**					
No	3991	1091730	3.66	1.00	1.00
Yes	71	6219	11.4	3.17(2.51, 4.01)***	1.88(1.48, 2.38)***

Rate, incidence rate, per 1,000 person-years; Crude HR, relative hazard ratio.

Adjusted HR,Variables found to be significant (p<0.05) in the univariate Cox model were then included in the multivariate Cox model; adjustedHR, denotesmultivariate Cox model including age, sex, and comorbidities of diabetes, hypertension, stroke, CAD, head injury, depression, and epilepsy; CAD denotes coronary artery disease; PY denotes person-years.

**p<0.01,

***p<0.001

Table 3 shows the incidence of dementia stratified by age, sex and comorbidity and Cox model measured HR for patients with IBS compared those without IBS. Except for patients aged 49 years and younger, the patients with IBS were significantly associated with an increased risk of dementia compared with the patients without IBS in two age groups (age 50–64 years, aHR = 1.34, 95% CI = 1.12–1.60; age, >65 years, aHR = 1.24, 95% CI = 1.14–1.34), both female(aHR = 1.16, 95% CI = 1.05–1.28) and male (aHR = 1.37, 95% CI = 1.24–1.52), and patients with(aHR = 1.43, 95% CI = 1.12–1.68) and without comorbidity (aHR = 1.29, 95% CI = 1.20–1.40).

**Table 3:** Incidence of dementia stratified by age, sex and comorbidity and Cox model measured hazard ratiofor patients with irritable bowel syndrome compared those without irritable bowel syndrome.

	Irritable bowel syndrome							
	No			Yes				
Variables	Event	PY	Rate	Event	PY	Rate	Crude HR(95% CI)	Adjusted HR(95% CI)
**Age, years**								
≤ 49	51	452941	0.11	21	115878	0.18	1.60(0.96, 2.66)	1.20(0.71, 2.04)
50–64	427	242049	1.76	172	60770	2.83	1.60(1.34, 1.91)***	1.34(1.12, 1.60)**
65+	2503	180398	13.9	888	45915	19.3	1.39(1.29, 1.50)***	1.24(1.14, 1.34)***
**Sex**								
Female	1579	471358	3.35	527	119769	4.40	1.31(1.19, 1.45)***	1.16(1.05, 1.28)**
Male	1402	404029	3.47	554	102793	5.39	1.55(1.41, 1.71)***	1.37(1.24, 1.52)***
**Comorbidity**								
No	730	601117	1.21	172	123511	1.39	1.14(0.97, 1.35)	1.43(1.21, 1.68)***
Yes	2251	274270	8.21	909	99052	9.18	1.11(1.03, 1.20)**	1.29(1.20, 1.40)***

Rate, incidence rate, per 1,000 person-years; Crude HR, relative hazard ratio.

Adjusted HR, Variables found to be significant (p<0.05) in the univariate Cox model were then included in the multivariate Cox model; adjusted HR denotes multivariate Cox model including age, sex, and comorbidities of diabetes, hypertension, stroke, CAD, head injury, depression, and epilepsy; CAD denotes coronary artery disease; PY denotes person-years.

**p<0.01,

***p<0.001

Table 4 shows the HRs of non-AD dementia and AD in association with gender, age, IBS and dementia-related comorbidities in univariate and multivariate Cox regression models. Compared with the non-IBS cohort, the patients with IBS were 1.76-fold more likely to develop AD (95% CI = 1.28–2.43) and exhibited a significantly higher risk of non-AD dementia (aHR = 1.24, 95% CI = 1.15–1.33). However, IBS may be less influential than other risk factors, such as head injury(non-AD dementia: aHR = 1.51, 95% CI = 1.28–1.77; AD; aHR = 2.22, 95% CI = 1.16–4.22) and depression (non-AD dementia: aHR = 1.88, 95% CI = 1.68–2.11; AD; aHR = 1.83, 95% CI = 1.06–3.14), in contributing to the development of non-AD dementia and AD.

**Table 4:** Hazard ratios of non- Alzheimer's disease dementia and Alzheimer's disease in association with gender, age, irritable bowel syndrome and dementia-related comorbidities in univariate and multivariate Cox regression models.

	Non-AD dementia				AD			
	Crude		Adjusted		Crude		Adjusted	
Variable	HR	(95% CI)	HR	(95% CI)	HR	(95% CI)	HR	(95% CI)
IBS	1.41	(1.31, 1.51)***	1.24	(1.15, 1.33)***	1.89	(1.38, 2.58)***	1.76	(1.28, 2.43)***
Gender (Female vs Male)	1.09	(1.02, 1.16)**	0.97	(0.91–1.03)	1.12	(1.11, 1.14)***	1.12	(1.11, 1.14)***
Age, years	1.12	(1.12, 1.13)***	1.12	(1.11, 1.12)***	1.08	(0.80, 1.44)	0.95	(0.71–1.27)
**Baseline comorbidities (yes vs no)**								
Diabetes	3.41	(3.14, 3.69)***	1.35	(1.25,1.47)***	3.68	(2.56, 5.29)***	1.63	(1.12, 2.36)***
Hypertension	6.32	(5.90, 6.76)***	1.35	(1.25,1.46)***	5.09	(3.74, 6.92)***	1.13	(0.80, 1.60)
Stroke	6.15	(5.57, 6.78)***	1.52	(1.38, 1.69)***	3.71	(2.15, 6.41)***	0.96	(0.55, 1.68)
CAD	4.68	(4.39, 4.99)***	1.13	(1.05, 1.21)***	3.63	(2.66, 4.94)***	0.88	(0.63, 1.23)
Head injury	2.42	(2.06, 2.84)***	1.51	(1.28, 1.77)***	3.32	(1.76, 6.29)***	2.22	(1.16, 4.22)***
Depression	2.67	(2.39, 2.99)***	1.88	(1.68, 2.11)***	2.55	(1.50, 4.33)***	1.83	(1.06, 3.14)***
Epilepsy	3.23	(2.55, 4.10)***	1.96	(1.54, 2.50)***	1.98	(0.49, 7.96)	-	-

Crude HR, relative hazard ratio;

Non-AD dementia Adjusted: Variables found to be significant (p<0.05) in the univariate Cox model were then included in the multivariate Cox model; adjusted HR denotes multivariate Cox model including age, sex, and comorbidities of diabetes, hypertension, stroke, CAD, head injury, depression, and epilepsy; AD denotes Alzheimer's disease; IBS denotes irritable bowel syndrome; CAD denotes coronary artery disease.

AD Adjusted: Variables found to be significant in the univariate Cox model (p<0.05) were then included in the multivariate Cox model, adjusted HR denotes multivariate Cox model including age, sex, and comorbidities of diabetes, hypertension, stroke, CAD, head injury, and depression; AD denotes Alzheimer's disease; IBS denotes irritable bowel syndrome; CAD denotes coronary artery disease.

**p<0.01,

***p<0.001

## Discussion

According to our research, this population-based study is the first to assess the association between IBS and subsequent dementia. The explanatory power of our statistical analysis is supported by the 12-year observation period and large national database, containing a representative cohort of 1 000 000 enrollees inTaiwan’s NHI program. Moreover, the literature investigating the relationship between IBS and dementia in humans is limited by small samples. Cognitive impairment is typically confirmed according to brain morphology assessment by using magnetic resonance imaging or cognitive ability assessment by using questionnaires, glucocorticoids analysis, or models for neuropsychological testing. Our study is the first epidemiological study involving humans to demonstrate a correlation with an increased risk of dementia following IBS diagnosis [12–15].

Taiwan’s NHI program, which provides access to affordable healthcare facilities, may have contributed to the overestimation of the prevalence of IBS in our study. However, we observed no sex difference insusceptibility to IBS between the IBS and non-IBS patients in this study, which supports the findings of previous studies investigating IBS in Asian populations, including those in Taiwan [3, 16]. In addition, we found no evidence that patients aged<50 years are particularly vulnerable to IBS development. By contrast, most epidemiological studies in Western countries have shown that IBS is more prevalent among women, particularly those who are <50 years old [17]. Estrogen might explain the susceptibility of female and younger patients to IBS in Western countries because it can increase the severity of visceral hyperalgesia and inhibit colonic motility [18,19]. Nevertheless, in addition to hormone factors, neuropsychological factors might also affect the clinical symptoms of IBS.

Our results show that compared with the patients in the non-IBS cohort, those in the IBS cohort tended to have more comorbidities, namely diabetes, hypertension, stroke, CAD, head injury, depression, and epilepsy. However, although the prevalence of health-care-related illnesses was higher among the patients with IBS, the risk of dementia remained higher in this cohort than in the non-IBS cohort following adjustment for diabetes, hypertension, stroke, CAD, head injury, depression, and epilepsy. Our study supports that the IBS cohort had more health-care-related illnesses. The reason for more prevalence of health-care-related illnesses in IBS patients remains unclear, but the literature has suggested that IBS patients are more vulnerable to psychosocial stress to have more medical consultations for GI symptoms or physical comorbidities [20–22]. The underlying physiological mechanism responsible for more comorbidities in patients with IBS also remains unclear, although a previous study postulated that alterations in plasticity, neurogenesis, catecholaminergic neurotransmission, and gut microbiota are potential mechanisms [23].

In the present study, dementia incidence increased with age, was higher among men, and tended to coincide with a higher comorbidity incidence. The patients >50 years demonstrated a greater risk of dementia, increasing progressively with age from 13.0 (50–64-years) to 85.5 (≥65 years). However, the prevalence of dementia-associated comorbidity might have been underestimated because patients with dementia tend to have limited access to medical services. The reasons for the higher risk of comorbidity with dementia may include low accessibility to medical services because of poor communication, inadequate nursing care or intake, locomotion impairment or psychiatric disturbance following neurodegeneration, and dementia caused by comorbidity [24].

The association between IBS and dementia in our study may be due to their shared pathophysiological mechanisms. First, the mechanisms associated with IBS are complex and may include environmental and host factors [1]. Environmental factors include early life stressors, food intolerance, antibiotics, and enteric infection; whereas, host factors include altered pain perception, dysbiosis, increased gut permeability, activation of gut immune system, visceral hypersensitivity, and altered brain-gut interaction. On the whole, an abnormal bidirectional interaction through the brain—gut axis has been proposed as one of the possible mechanisms to explain the correlation between IBS and deregulated brain or behavior performance. The byproduct of gut dysbiosis can access the brain through cytokines released from mucosal immune cells, gut hormones released from enteroendocrine cells, or afferent neural pathways of the ENS influencing brain function and behavior. In addition, stress and emotional disturbance can cause gut dysbiosis and affect the gut physiology through the release of stress hormones or regulation of neuroendocrine transmitters [25]. Second, bacterial colonization and gut inflammation are detected by the afferent component of the vagus nerve, and the efferent response is communicated via vagal anti-inflammatory output [26,27]. Animal studies have suggested that the vagus nerve is the main route for transmitting cytokine signals to the brain, inducing neuroinflammation and potentiating neurotoxic substances [28,29]. IBS may increase lipopolysaccharide, complement factor, acute-phase protein, and proinflammatory cytokine levels in the brain, which have been implicated in the pathogenesis of dementia [30,31, 32]. Third, the hippocampus and cerebral cortex are regarded as the main foci for amyloid deposition following neuroinflammation, exacerbating cognitive dysfunction and increasing vulnerability to dementia [33, 34]. Moreover, the cerebellum is strongly related to cholinergic function and astroglial activation after neuroinflammation in the cerebellum, contributing to cognitive impairment in IBS [35].

Furthermore, the association between IBS and dementia in this study might be caused by shared risk factors since the prevalence of risk factors for dementia was higher in the patients with IBS than in those without IBS. However, we have adjusted for the possible dementia-associated factors, and it is reasonable to conclude that the correlation between an increased risk of dementia and IBS was likely to be caused by the effects of IBS status (Table 2). Our results indicate that the effect of IBS on the relative risk of dementia peaks between age 50–64 and then decreases after age 65, which might be due to increased prevalence of other dementia-associated risk factors in older patients. The results from our subgroup analyses with stratification of comorbidities could validate our results. We could not ascertain the temporal association between the occurrence of IBS and dementia. However, our results suggest the risk of dementia increases with the increment of follow-up duration, rather than in the immediate days, in patients with IBS. Overall, our results indicate a possible causal association between IBS and dementia, and suggest that IBS is a risk factor for dementia. However, our results showed that IBS may be less influential than those traditional dementia-associated risk factors such as aging, diabetes, hypertension, stroke, head injury, depression, and epilepsy, in contributing to the development of dementia.

Several limitations were observed during the course of our study. First, we were concerned about the validity of the IBS and dementia diagnoses among the patients enrolled in our study. However, the ICD-9-CMdiagnostic codes made based on Rome criteria for IBS and Diagnostic and Statistical Manual of Mental Disorders 4^th^ edition(DSM-IV)for dementia are widely used in Taiwan, and the Bureau of National Health Insurance requires medical experts to regularly audit insurance claims in Taiwan. The NHI program has been monopolistically operated by the Taiwan government since 1995, and the accuracy of diagnosis were judged and determined by related specialists and physicians. The hospitals and doctors would be punished to pay a lot of penalty if they were judged to be incorrect in making the diagnoses. Therefore, the diagnoses of osteoporosis and dementia coded in this study should be correct and reliable. To ensure accurate diagnosis, we included only cases in which medical care was administered for IBS and dementia, either through outpatient visits or hospitalization, at least three times. Second, bias may have resulted from patients with IBS seeking medical care more frequently compared with patients without IBS, increasing the likelihood of dementia or comorbidity diagnosis during follow-up. Moreover, certain potentially confounding factors could not be verified because the NHIRD does not contain detailed information related to the lifestyle, socioeconomic status, and the family history of patients. However, after adjustment for the comorbidities of diabetes, hypertension, stroke, CAD, head injury, depression, and epilepsy, the risk of dementia among the patients with IBS remained higher than that among the patients in the non-IBS cohort. Finally, patients who are not enrolled in the NHI program were not included in our study population. However, Taiwan’s NHI program provides coverage for over 99% of the national population.

In conclusion, this nationwide population-based cohort study shows that IBS is associated with a higher risk of dementia and this effect is obvious only in patients who are ≥50 years old. Both IBS and dementia are independently related to a higher prevalence of healthcare-related illnesses. No difference in sex was observed between the IBS and non-IBS cohorts.
